# Core Concepts: Self‐Controlled Designs in Pharmacoepidemiology

**DOI:** 10.1002/pds.70071

**Published:** 2025-01-13

**Authors:** Sophie H. Bots, Jeremy Brown, Angel Y. S. Wong, Ivonne Martin, Ian Douglas, Olaf H. Klungel, Anna Schultze

**Affiliations:** ^1^ Division of Pharmacoepidemiology and Clinical Pharmacology, Utrecht Institute for Pharmaceutical Sciences Utrecht University Utrecht Netherlands; ^2^ Department of Epidemiology, Harvard T.H. Chan School of Public Health Harvard University Cambridge Massachusetts USA; ^3^ Department of Non‐communicable Disease Epidemiology London School of Hygiene and Tropical Medicine London UK; ^4^ Department of Data Science and Biostatistics, Julius Center for Health Sciences and Primary Care University Medical Center Utrecht Utrecht Netherlands

**Keywords:** case‐crossover design, self‐controlled case series, self‐controlled study designs

## Abstract

One of the key challenges in pharmacoepidemiological studies is that of uncontrolled confounding, which occurs when confounders are poorly measured, unmeasured or unknown. Self‐controlled designs can help address this issue, as their key comparison is not between people, but periods of time within the same person. This controls for all time‐stable confounders (genetics) and in the absence of time‐varying confounding negates the need for an external control group. However, these benefits come at the cost of strong assumptions, not all of which are verifiable. This review briefly introduces the reader to different types of self‐controlled study designs, their terminology and highlights key publications through an annotated reference list. We include a practical description of how these designs can be implemented and visualised using recent examples, and finish by discussing recent developments. We hope this review will serve as a starting point for researchers looking to apply self‐controlled designs in their own work.

**Summary**

- Self‐controlled study designs generally include only individuals who experience the outcome, and the key contrast involves a comparison of a time period of hypothesised increased risk of exposure or outcome (focal window) to one or more reference time periods (referent window) within the same person.
- Self‐controlled study designs can be outcome‐anchored, in which the frequency of exposure is compared between focal and referent windows defined relative to an outcome event (such as the case‐crossover study); or exposure‐anchored, in which frequency of the outcome is compared between focal and referent windows defined relative to an exposure (such as the self‐controlled case series).
- The key advantage of self‐controlled studies is that potential confounding factors which are stable across the focal and referent windows are implicitly controlled for, irrespective of whether or not they are known or measured. They also allow for more efficient data collection, and computational efficiency, compared to traditional between‐person study designs.
- All self‐controlled designs rely on strong assumptions, which need to be carefully evaluated. They are particularly useful for research questions where there is a transient exposure, an acute outcome and either unmeasured confounding is a key concern or a suitable control group is difficult to define.

## 1 Introduction

Most analytical studies in pharmacoepidemiology compare the risk of an outcome between individuals who are and are not exposed to a certain medication, typically using either a cohort or a case–control design [1]. In contrast to cohort and case–control studies, self‐controlled study designs compare different time periods within the same person. This means that individuals act as their own controls and all confounders that do not vary over the observation period (e.g. genetic factors) are implicitly controlled for, irrespective of whether or not they are measured or known. Self‐controlled designs also negate the need to identify or collect data on a separate control group, and can be more statistically efficient, if the resources saved on not extracting data for non‐cases enables the inclusion of more cases [2, 3]. In this review, we provide a brief introduction to different self‐controlled study designs for pharmacoepidemiologists. Our aim is to complement existing high‐quality reviews [4, 5] by integrating new terminology and visualisation standards [6, 7], highlighting recent developments, and providing a roadmap of existing resources through an annotated reference list.

## 2 Key Features of Self‐Controlled Studies

All self‐controlled studies share some key features, although the terminology used to describe these has historically differed between the different designs. Table 1 summarises relevant terminology, following a recent effort to harmonise terminology which also proposed the term Self‐controlled Crossover Observational PharmacoEpidemiologic (SCOPE) studies to refer to studies using self‐controlled designs [6].

**TABLE 1:** Key features of self‐controlled studies.

Key feature	Definition	Alternative names
Anchor	A point in time relative to which all design features in a self‐controlled study are defined. The anchor is either the exposure or the outcome depending on the type of self‐controlled design. A study can have more than one anchor, for example if there are multiple outcomes within the same person.	
Exposure‐anchored	Self‐controlled studies in which key design features are defined relative to the timing of one or more exposure dates.	
Outcome‐anchored	Self‐controlled studies in which key design features are defined relative to the timing of one or more outcome dates.	
Observation period	Calendar time period during which individual cases are observed. In general, this will include time when it is possible for both the outcome and exposure to occur. The observation period can include time where the outcome (outcome‐anchored) or exposure (exposure‐anchored) cannot occur if the usual risk in this period is representative of the usual risk in the period where the outcome or exposure is present.	Observation windows
Study period	Calendar time period during which eligible cases will be identified. The study period does not need to be equal to the observation period and can span multiple years to ensure sufficient cases are included in the study.	
Focal window	Period of time, within a person, during which the risk of either an outcome or exposure is hypothesised to be heightened. The focal window should be defined based on the underlying hypothesised biological or pharmacological effects that are being studied, for example, reflecting varying lengths of exposure to a particular drug.	Risk window/period Hazard period
Referent window	Period of time, within a person, during which the risk of either an outcome or exposure is hypothesised to reflect the usual incidence.	Control window/period Baseline period
Transition window	Period of time that should not be considered as part of either a focal or the referent window because the risk of the outcome or exposure does not reflect either the hypothesised increased risk or the usual incidence. This includes lingering effects of the exposure on the outcome (wash‐out), a delay between administration of exposure and the start of its effects (induction), a delay between an outcome and subsequent exposure (pre‐exposure) and periods of lower risk due to data quality or healthcare system factors (lag).	Pre‐exposure window/period Wash‐out window Induction window Lag window
Covariate assessment window	Period of time, prior to the earliest referent or focal window, during which baseline covariates are assessed. In self‐controlled designs, eligibility criteria are typically applied either at the anchor date, or at the start of the study period if they are nested within a larger cohort. A minimum covariate assessment window (for example 1 year) may be added as an eligibility criteria. Time‐varying covariates (e.g. age, calendar time) are assessed throughout the study period in exposure‐anchored designs. Follow‐up time is then split into segments defined by periods (e.g. months) of this covariate. In theory, other time‐varying covariates could be adjusted for in the same way, but caution is needed to ensure that the time‐varying does not also act as a mediator, in which case adjustment would be inappropriate.	

*Note:* As the vocabulary for self‐controlled designs has evolved naturally over the years and attempts to unify inconsistencies are relatively recent [6], a column with alternative names used in literature has been included.

### 2.1 Study Design and Analysis

All self‐controlled designs have one or more *anchors*; points in time relative to which all other design features are defined. Self‐controlled designs then divide the observation time into periods of hypothesised increased risk (*focal windows*) and comparison periods (*referent windows*) relative to the anchor(s). Additional time periods, *transition windows*, that should not be considered as part of either a focal or the referent window, may also be defined (Table 1). Taken together, these windows are sometimes referred to as *observation windows* or the *observation period*. This should be differentiated from the *study period*, which defines the calendar time period during which eligible cases are identified. For example, when studying the effect of a vaccine given during the second year of life, the observation period for each individual is the second year of life, whereas the study period could span several years to include a sufficient number of cases.

Usually, the observation period should include time when it is possible for both the outcome and exposure to occur. However, in some instances it might be valid to sample referent windows during which the outcome (in outcome‐anchored studies) or the exposure (in exposure‐anchored studies) could not occur [2]. As the aim of the referent window is to provide an estimate of the ‘usual’ frequency of either the exposure or outcome, non‐risk time periods can be used to estimate this provided that they are representative of periods of time during which the relevant event could occur [8, 9]. For example, in an exposure‐anchored study of a vaccine and an adverse event, researchers may choose to start the study period before the roll‐out of the vaccine. Eligibility criteria in self‐controlled designs are often applied either at the anchor date, or at the start of the study period if they are nested within a larger cohort. The decision of when to apply eligibility criteria may be taken for pragmatic rather than scientific reasons: when conducting primary data collection based on the sampling of cases, assessing eligibility at the outcome date may be easy to implement. However, when adding a self‐controlled case series to an ongoing cohort study, assessing eligibility at the start of the study period may allow researchers to use the same data cut for both analyses.

The final feature which all self‐controlled studies share is that they condition on the individual during the analysis: that is, they are analysed in strata defined by the individual such as by using conditional Poisson or logistic regression. This is of key importance: it is what enables the control of time‐stable confounders.

### 2.2 Advantages and Assumptions

The key advantage of self‐controlled designs compared to between‐person designs is the control of time‐stable confounders. Removing the need for a separate unexposed control group is also useful in scenarios where a suitable control group is difficult to identify, for example, due to large differences between exposed and unexposed individuals or vaccination campaigns where the exposure quickly becomes universal. In cases where primary data collection is done, collecting data on only cases is also likely to be quicker and cheaper than collecting additional data on a control group. Researchers do still need to be aware of the risk of time‐varying confounders such as age and seasonality, which can be minimised through the use of concurrent controls or statistical adjustment [2, 3, 10, 11, 12]. Methods for handling time‐varying confounders are elaborated on in the design‐specific sections.

Self‐controlled designs also share some important assumptions. First, they generally require that exposures should be transient. An important nuance here is that permanent changes in exposure status can still be studied as long as the effect of the exposure on the outcome is transient [2], which may be the case even if the exposure itself is permanent. For example, vaccination is a permanent change in exposure status (from unvaccinated to vaccinated), but it is only expected to increase the risk of side effects (like injection site pain) for a short period directly after receipt of the vaccine. The underlying hypothesis should be that the exposure affects risk of the outcome in the short‐term. Although there are examples of self‐controlled studies evaluating long‐term medication use (so‐called persistent or chronic use), for example, the effect of antipsychotic medications on fracture risk [13], caution is warranted and this is discussed further below.

Second, self‐controlled designs require that the timing of the outcome of interest is correctly identified, and they are therefore best suited to study *acute events* or events with abrupt onset recorded with a precise date. The precision of the outcome measurement should match the research question; if you are interested in whether an outcome occurs minutes after exposure then you must be able to identify the exact minute the outcome occurred [2]. Selection bias may be introduced if the probability of experiencing an event during a focal window compared to a reference window differs between included and excluded exposed cases. For example, if adverse events occurring shortly after vaccination are more likely to be recorded than those occurring before or longer after, individuals with events in focal windows are more likely to be included than those with events at other moments. This would result in an overestimation of the association between vaccination and the adverse event. Measurement error in either the exposure or outcome timing is a threat to the validity of a self‐controlled analysis, as it means that focal and referent windows may be specified incorrectly and events attributed to the wrong time window.

Finally, because self‐controlled designs are case‐based, they *do not directly estimate absolute risks*. These can be indirectly estimated, provided there is some denominator information available; alternatively published formulas based on relative measures of effect can be used to calculate an attributable fraction [14, 15]. There is limited guidance on how to calculate absolute measures of effect for self‐controlled studies, and this should be done with particular caution if denominator information is derived from external sources [16].

### 2.3 Related Study Designs

There are a number of related designs that are not all considered self‐controlled study designs, either because they still rely on comparisons between individuals to make inferences despite including only cases, or because their aim is signal detection [17]. These include the sequence symmetry analysis developed for medication signal detection [18], the self‐controlled tree scan statistic [19] and the case‐referent method developed for vaccine safety assessments [20, 21].

## 3 Outcome‐Anchored Self‐Controlled Designs

### 3.1 Case‐Crossover Design

**FIGURE 1:**
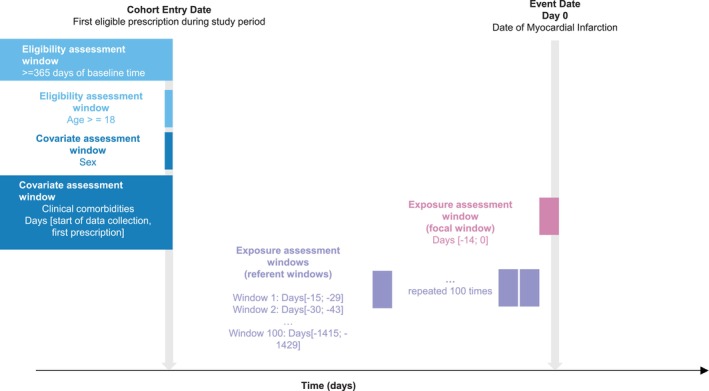
Illustration of a case‐crossover study to evaluate the association between clarithromycin and myocardial infarction (Wong et al. [26]).

The case‐crossover (CCO) design was developed as an alternative to the case–control design [22]. The design uses the timing of the exposure relative to the outcome to make inferences [2]: intuitively, this can be thought of as evaluating how likely it is that, given that the outcome has occurred at this specific point in time, the exposure happened just before it? The CCO is outcome‐anchored, with a focal window just before the outcome and one or more referent window at earlier time points. The probability of exposure is then compared during focal and referent windows to estimate an odds ratio, using either conditional logistic regression or the Mantel–Haenszel method stratified on the individual. In general, the CCO's efficiency increases with increasing number of referent windows [23], although there is a trade‐off, as a greater number of referent windows risks exacerbating bias due to time‐varying confounding if referent windows get further away from the anchor [2]. Researchers should be aware that using conditional logistic regression can introduce bias if more than one control period is used due to an issue called within‐subject exposure dependency (see [24] for more details). This could be removed by using the Mantel–Haenszel method [25]. An example of a CCO is provided in Box 1, and the design is illustrated in Figure 1.

BOX 1 **Example case‐cross over study: clarithromycin and myocardial infarction.**

Wong et al. used a case‐crossover design to study the association between use of clarithromycin, an antibiotic which is used for short periods of time, and myocardial infarction using data from the Clinical Data Analysis and Reporting Systems database in Hong Kong [26]. To implement the study, they first selected individuals who had experienced a myocardial infarction between 1 January 2003 and 31 December 2012. The eligibility criteria (age ≥ 18 years and ≥ 365 days of data availability) were assessed at the first prescription date as the study was nested within an accompanying cohort study. A single focal window of 14 days prior to the date of MI, and 100 referent windows of the same length were defined, as illustrated in Figure 1.

To analyse the data, each patient contributed a maximum of 101 rows: 1 for the focal window, and 100 for the reference windows. The minimum dataset necessary for analysis contained three columns: one for patient ID, one identifying whether the row represented a focal window and one identifying whether the exposure occurred in the relevant window. The case‐crossover odds ratio was estimated by fitting a conditional logistic regression model, with exposure as the dependent variable and focal window as the independent variable.

A core assumption of the CCO is that the probability of exposure in the referent window represents that in the focal window under the null hypothesis of no exposure effect [11]. This has some important implications. Firstly, it implies that there should be *no strong population‐level time trends in the exposure*. If the probability of exposure increases over time, exposure would be more likely in focal than reference windows thus biasing the effect estimates upwards. A bi‐directional CCO using referent windows both before and after the outcome can control potential exposure time trends, but this requires the additional assumption that the outcome should not affect future exposure probability [2, 27]. Secondly, it means that results from a CCO will be biased if the focal and referent window are not comparable, for example, that is, there should be *no time‐varying confounding*. Such time‐varying confounders can be adjusted, assuming they are measured during both referent and focal windows [9], and the timing of the exposure and confounder do not completely overlap.

Pharmacoepidemiologists should be aware of a number of additional important considerations when interpreting results from a CCO. Firstly, there is a risk of ‘carry‐over’ effects if the effect of a medication is not transient, blurring the effects between the focal and referent window. A transition (wash‐out) window is typically placed between the referent and focal windows to account for this [28]. Upward bias might also be introduced when studying drugs that should be taken chronically (‘persistent use’) in a CCO because it only includes individuals with discordant exposure status. In a theoretical situation where all people use the medication persistently, the only individuals with such discordant exposure would be those who have the side effect shortly after treatment initiation and are thus exposed in the focal window but unexposed in the referent window (because drug use should be continuous in the absence of the side effect) [29, 30]. In the real‐world, there will always be both transient and persistent users, and the mix of these subpopulations can drive an upward bias. We refer readers to [30] for a more in‐depth discussion of persistent user bias. Recent work suggests using referent time points instead of windows may alleviate this issue, allowing the study of persistent use in a CCO [31]. Finally, as in other study designs, these designs are also at risk of selection bias if the exposure distribution amongst included cases differs from that amongst all cases in the target population [9] and from bias due to misclassification.

### 3.2 Variants and Extensions of the Case‐Crossover Design

**FIGURE 2:**
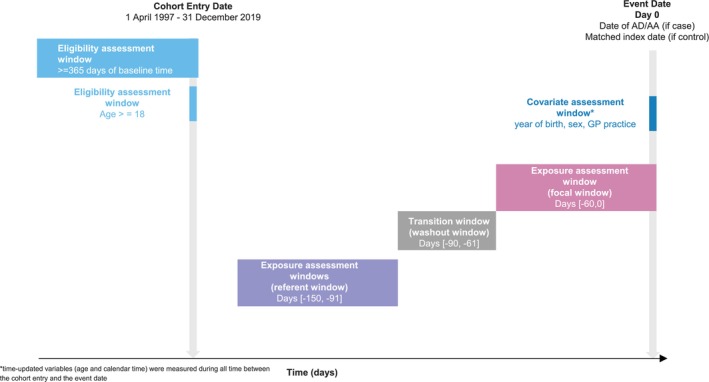
Illustration of a case‐time‐control study to evaluate the association between FQ and AD/AA (Brown et al. [34]).

The case‐time‐control (CTC) study is an extension of the CCO study developed to account for potential exposure time trends caused by for example progression of underlying conditions or changes in prescription practices over time [12]. The CTC uses a control group of individuals without the outcome to quantify, and correct for, any time trend in the exposure. Briefly, each case is matched to a control, assigned the event date of the case, and a referent and focal windows are defined relative to the assigned event date. The odds of exposure in focal and referent window amongst controls is then compared to calculate a ‘trends odds ratio’. By dividing the CCO odds ratio with the trends odds ratio estimated in controls, an odds ratio can be derived adjusted for time trends. There is also some evidence suggesting that CTC may be less biased than the CCO when studying persistent users, although incorporation of a control group does not appear completely satisfactory in resolving this issue [30]. An important assumption of the CTC design is that the exposure time trend is the same amongst cases and controls: this may not hold true if cases are generally sicker and therefore more likely to use medication in the time period leading up to their event. Researchers can match on person‐level characteristics, including age, sex and disease risk score to make the control group, and thus ideally time trends, more similar to cases [32]. An extension of the CTC, the case–case‐time control design, involves using future cases as controls [33]. This has several proposed benefits: firstly, it avoids sampling an external control group of non‐cases, and secondly, it might provide a more comparable estimate of exposure time trends, as future cases may be more similar to current cases than non‐cases. An example of the CTC is provided in Box 2, and the design is illustrated in Figure 2.

BOX 2 **Example case‐time‐control study: fluoroquinolone and aortic aneurysm or dissection.**

Brown et al. used a case‐time‐control design to assess the association between the use of fluoroquinolones, a class of antibiotic typically used for short periods of time, and hospitalisation for aortic aneurysm or dissection (AA/AD) [34]. Data sources were UK electronic health records from the Clinical Practice Research Datalink (Aurum) and GOLD databases. Incident cases of hospitalisation for AA/AD were identified during an eligibility window that started at the latest of 1st of April 1997, one year after registration at current practice, 18th birthday and 1 year after practice data was deemed to be of research quality by the data provider. The eligibility window ended at the earliest of 1st December 2019, last data collection at practice, death or patient transfer out of practice. The focal window was defined as the 60 days prior to the outcome, and the referent window was 90–150 days before the outcome (following a 30‐day transition window [wash‐out window]). Controls, matched on index date, year of birth, sex and primary care practice, were used to calculate an odds ratio adjusted for time trends in exposure (Figure 2).

To estimate an odds ratio adjusted for exposure trends, a conditional logistic regression model was fitted with an interaction term between exposure and case status [12]. A simple ratio approach was taken to estimate an active comparator odds ratio relative to other comparator antibiotics (cephalosporin, trimethoprim and co‐amoxiclav) [10].

The CCO can also incorporate active comparators, which can help address concerns around time‐varying confounding [10] and protopathic (reverse causality) bias [35]. Finally, the CCO design has been extended to study drug–drug interactions with a six‐parameter model that allows the order of drug initiation to be differentiated: a detailed description of how to use the design for this purpose is provided by Bykov et al. [29].

## 4 Exposure‐Anchored Self‐Controlled Designs

### 4.1 Self‐Controlled Case Series

The self‐controlled case series (SCCS) was developed to investigate adverse events following vaccination [36], although it has since been applied more widely [37] to study the safety of medicines, devices and medical procedures. The SCCS aims to answer an underlying research question about the timing of the outcome relative to the exposure: given that the exposure takes place at a certain point in time, how likely is it that the outcome occurs shortly after? [3].

**FIGURE 3:**
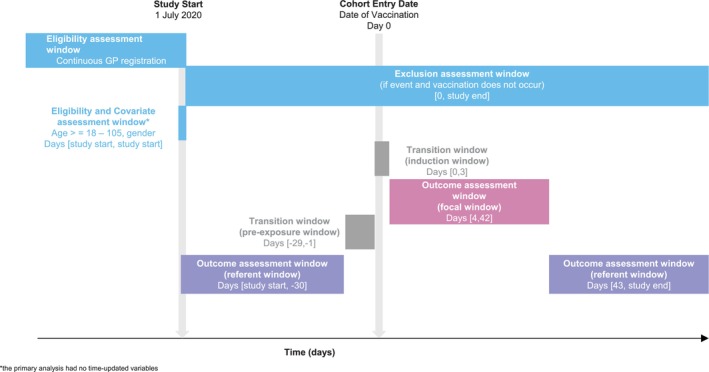
Illustration of a self‐controlled case series to evaluate the association between COVID‐19 vaccines and GBS (Walker et al. [39]).

The SCCS defines referent and focal windows relative to the exposure within the pre‐specified observation period. Typically, all time during the observation period outside the focal window is used as the referent window. In other words, the observation period should not end when the outcome occurs, making the SCCS a bi‐directional design [4]. The length of the observation period in an SCCS can vary from relatively short to several years, and the difference in time contributed by the focal and referent windows is accounted for by including the length of the window as an offset during analysis: typically using conditional Poisson regression to estimate a rate ratio. Because the SCCS is bi‐directional, it does not suffer from the same persistent user bias as the CCO when studying chronic medication use. However, studying chronic compared to short‐term medication use in this design does still require specific consideration as the risk of time‐varying confounding is increased. Power in an SCCS depends both on the number of cases included, and the ratio of referent to focal time; power calculations SCCS can be conducted using both Stata and R [15, 38]. An example of the SCCS is provided in Box 3, and the design is illustrated in Figure 3.

BOX 3 **Example self‐controlled case series: COVID‐19 vaccines and GBS.**

Walker et al. used a self‐controlled case series design to study the association between the first dose of the COVID‐19 vaccine and the risk of neurological safety events, including GBS using data from OpenSAFELY‐TPP [39]. Although vaccination is not a transient exposure, the increase in the risk of GBS after vaccination was hypothesised to be transient. Adults with at least 1 year of continuous GP registration on 1 July 2020 were included, and those with missing age, sex, postcode or pregnancy were excluded. The end of the study period was 7 July 2021. After each COVID‐19 vaccine, a focal window of 4–42 days was added. The first 4 days after vaccination was considered as a transition window (induction window) (as development of the outcome was considered biologically impossible), and the 28 days before vaccination was used as another transition window (pre‐exposure window) (Figure 3). The primary analyses were unadjusted for calendar time, as minimal trends in these outcomes were expected over the study period.

For each individual, a row for each relevant time period was created, and a rate ratio calculated through conditional Poisson regression using the natural log of the length of each time window as an offset.

The SCCS has three key assumptions that must be met for the design to give valid estimates [3, 37, 40]. First, *outcomes need to be either independently recurrent, or rare*, where rare is defined as an incidence of < 10% in the whole cohort during the study period [41]. If the outcome is not independently recurrent, the easiest solution is to only study the first event that occurs within the study period [37] provided that this is rare, or to use analytical extensions [42, 43]. Second, *exposure should be event‐independent*; that is, the probability of exposure should not be affected by the outcome of interest. This assumption is particularly important in pharmacoepidemiology, as people who experience a safety outcome of interest may delay or be contraindicated for further treatment.

Historically, limited violations of this assumption have been handled by defining a transition window (pre‐exposure period) right before the exposure, which is excluded from the referent window [37]. The underlying reasoning is that if the outcome decreases the probability of exposure, the probability of having an outcome will be lower right before exposure. By removing this time from the referent window, we ensure the remaining time reflects the ‘usual’ frequency of the outcome. However, recent work [44] has shown that the ability of pre‐exposure windows to correct for bias can vary depending on the way in which the outcome affects exposure. In situations where there is only a short‐term delay to the exposure following the event, the extent of potential bias—counterintuitively—does not depend solely on the length of the delay, but also the length of the observation period, and when during the observation period most exposures occur. Specifically, bias can be introduced by short‐term delays if these result in exposures being delayed beyond the end of the observation period, with the result that some cases are excluded from the case series. For example, in a study of first COVID‐19 vaccine doses and venous thromboembolism (VTE), the occurrence of a VTE would be expected to delay receipt of the first dose. However, if the observation period is long—for example, spanning several years, such delays would not lead to ‘missing’ first doses due to the observation period coming to an end. In this situation, there is theoretically no bias despite the fact that the occurrence of the outcome causes the exposure to be delayed, and inclusion of a pre‐exposure window would be expected to underestimate the rate ratio. Further simulation studies to investigate the ability of pre‐exposure windows to correct for bias in the SCCS and its variants are needed: in the meantime, we recommend that researchers concerned about the violation of the assumption of event‐dependent exposures conduct sensitivity analyses using extensions of the SCCS that have been developed specifically to handle these situations [45].

Third, the *observation period should be event‐independent*; that is, the outcome of interest should not affect the length of the study period. This assumption is clearly violated when the outcome is death or has a high mortality rate. When studying a single exposure, violations of both the second and third assumption can be handled by starting follow‐up at the first exposure. Otherwise, extensions of the design need to be used [46].

The SCCS is also sensitive to time‐varying confounders, such as age or treatment indication. In situations where it is difficult to adjust for these, alternative methods such as the semi‐parametric SCCS [47, 48] can be considered.

### 4.2 Variants of the Self‐Controlled Case Series

**FIGURE 4:**
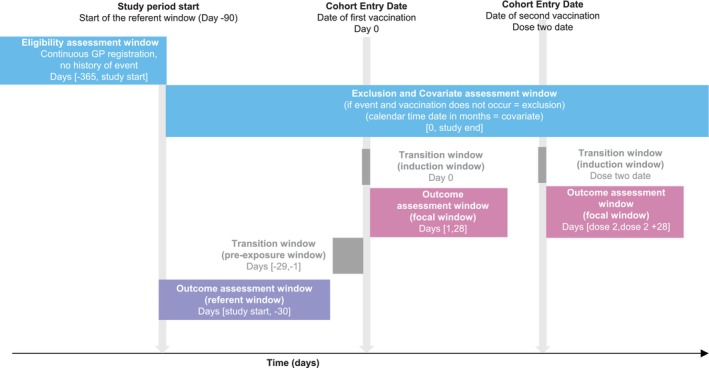
Illustration of a self‐controlled risk interval design to evaluate the association between COVID‐19 vaccines and myocarditis (Bots et al. [52]).

The self‐controlled risk interval (SCRI) is a variation on the SCCS, which specifies one or more discrete referent windows and disregards remaining follow‐up time during the study period [49]. Referent windows can be placed either before or after the exposure, or, in the case of multiple windows, both before and after the exposure (resulting in a bi‐directional design) [41]. The key benefit of the SCRI compared to the SCCS is that it is easier to operationalise in its simplest form, each individual contributes only two rows. In contrast, it is not unusual for the data management required for an SCCS to be more complex—particularly if season or age trends need to be taken into account. Existing comparisons of the SCCS and SCRI are scarce: but simulation studies indicate that their sensitivity to bias is similar [40, 50], and applied studies using both designs have found comparable results [51]. An example of the SCRI is provided in Box 4, and the design is illustrated in Figure 4.

BOX 4 **Example self‐controlled risk interval: COVID‐19 vaccines and myocarditis.**

Bots et al. used a self‐controlled risk interval design to study the association between COVID‐19 vaccination and the risk of myocarditis using data from five European databases [52]. The hypothesis was that COVID‐19 vaccination may increase the risk of myocarditis for a short time after vaccination. All vaccinated cases occurring between 1 September 2020 and the end of data availability were eligible for inclusion, exclusion criteria were less than 365 days of follow‐up before the start of the study period and a history of myocarditis. The date of the first vaccination was defined as Day 0, the referent window was placed before the first vaccination (day −90; −30) and a transition window (pre‐exposure window) (day −29; −1) was implemented to account for potential event‐dependent exposure. After each vaccination instance, a 28‐day focal window was defined (Figure 4). Analyses were adjusted for calendar time in 30‐day windows because the COVID‐19 pandemic was expected to introduce time trends in outcome incidence.

For each individual, a row for each relevant time period was created, and an incidence rate ratio was calculated through conditional Poisson regression.

There are several methodological extensions of the SCCS, designed to accommodate situations where key underlying assumptions may be violated, for example when the outcome of interest is death and thus exposure is no longer event‐independent. There is an extension based on estimating equations that can account for potential violations of the event‐dependent exposure assumption. It is readily implemented using the R package SCCS, although it does require that unexposed cases are included as it relies on the estimation of counterfactual exposure histories. This extension is also the main option available to researchers studying multiple exposures in the presence of violations of the assumption of event‐dependent exposures [53]. This extension can also be combined with proposed methods for handling event‐dependent observation periods like restricting the study to first events only [46]. Another variant is the so‐called ‘truncated SCCS’, these are equivalent to an SCRI with multiple post‐exposure referent windows, the length of which is determined by the anticipated vaccinations schedule between doses [54]. This extension can also accommodate violations of the assumption of event‐dependent exposures, and may be more straightforward to implement. Flexible versions of the SCCS that can accommodate dependency between events have also been developed, although software for its implementation is not yet widely available [43]. Finally, there are several approaches which allow researchers to handle time‐varying confounding. Although this can be accounted for analytically by splitting the follow‐up time according to the values of the time‐varying confounders and including them as covariates in the regression model, it may be challenging to accurately model these effects in case of small numbers. It is therefore recommended to include unexposed cases in the case series, as this allows for more accurate modelling of time‐varying confounders [15]. Active comparators can also be used for this purpose, if available [10].

## 5 Choosing a Self‐Controlled Study Design

Self‐controlled designs can either be an alternative or addition to a between‐person design, depending on the research question. As mentioned before, self‐controlled designs are most suited to study the acute effects of transient exposures. Although it is possible to study persistent or chronic exposures in exposure‐anchored self‐controlled designs, provided there is at least some unexposed follow‐up time, caution should be exercised [6]. The risk of time‐varying confounding increases with increasing length of follow‐up, and is particularly high if there is a systematic ordering of the focal versus referent windows, that is, if all focal windows occur before or after the referent windows. Self‐controlled designs are ill‐suited to study outcomes or exposures that are difficult to establish the timing of correctly, such as diseases with an unclear time of onset (endometriosis) and treatments that are taken ‘as needed’ (migraine medication), in which case a between‐person design is more appropriate. However, it seems self‐controlled designs have historically been underused in pharmacoepidemiology, with a systematic review identifying ‘missed opportunities’ for the application of self‐controlled case series in approximately 15% of pharmacoepidemiology studies in 2014 [55]. Worksheets to help researchers assess the suitability of a self‐controlled design in different situations are available [6].

Traditionally, self‐controlled designs have been presented as answering a slightly different question (‘why now’) compared to between‐person designs (‘why me’) [3]. However, recent work putting the CCO in a formal causal inference framework has shown that the CCO odds ratio can approximate the (causal) hazard ratio from a hypothetical randomised trial when a number of assumptions, largely corresponding to the informal assumptions listed in this paper, are met [56]. Of course, estimands from self‐controlled designs may not always correspond to those from similar between‐person designs due to subtle differences in treatment strategies considered, time periods covered, or the effect measure calculated. When using more than one design, it is important to consider these aspects to ensure that measures of effect derived from different designs can be fairly compared and contrasted. Despite these complexities we encourage researchers to consider whether a self‐controlled design may be a useful complement to ongoing cohort or case–control studies, as the use of more than one study design (each with their own distinct limitations and assumptions) is an important principle of triangulation of evidence [57].

Which self‐controlled study design is most suitable will also depend on the research question. In general, outcome‐anchored self‐controlled designs will be more sensitive to time trends in the exposure, and these may therefore not be suitable for the study of exposures where there are rapid time trends or changes in prescribing, such as vaccines. However, outcome‐anchored designs (provided that referent windows precede focal windows) do not make any assumptions around event‐dependent exposures and observation periods. This means that they may be more suited for the study of events with a high mortality rate or side effects which can result in contraindication. There are also important pragmatic considerations to take into account: in some instances it might not make a material difference if a SCCS or SCRI study design is used, but pragmatic considerations such as the time taken for post‐exposure follow‐up time to accrue might mean that one option is preferable to the other. In situations where the exposure has an indirect protective effect of the outcome—for example, COVID‐19 vaccines might be associated with a lower risk of cardiovascular outcomes due to their protective effect against SARS‐CoV‐2 infection—bi‐directional study designs or study designs using post‐vaccination referent windows may be more suited for studying the direct effects of the exposure. A study comparing the case‐crossover and self‐controlled case series found that both led to comparable conclusions in the absence of violations of the underlying assumptions [58], emphasising the importance of ensuring that researchers choose a self‐controlled design that is suited for their specific research question. This is in line with findings from a later simulation study comparing a case‐crossover and self‐controlled case series for the purposes of studying drug–drug interactions, which found that when no assumptions were violated, both designs were unbiased with the self‐controlled case series having slightly better precision [59].

## 6 Sensitivity Analyses for Self‐Controlled Study Designs

For self‐controlled studies, common sensitivity analyses involve varying the length of the reference and focal windows. For outcome‐anchored studies, these explore the presence of exposure time trends—for example, by plotting prescription trends over time. In exposure‐anchored studies, these explore assumptions around event‐dependent exposures: this might involve graphical assessments using exposure‐centred interval plots [40] or the application of methodological extensions described above.

Methods such as quantitative bias analysis (QBA) and negative controls have rarely been applied to self‐controlled settings, and researchers may find it more challenging to apply, as many tools and tutorials on these methods have not been developed with self‐controlled designs in mind. For example, summary‐level QBA for misclassification is typically based on the construction of contingency (2 × 2) tables [60]: these are less intuitive to construct for self‐controlled studies. We recently developed a simple framework adaptation of QBA for an SCRI based on the assumption that the SCRI compares observed and expected intervals in which both exposure and outcome are present, and modelling this assumption with and without confounders [61], but more research on the application of QBA in self‐controlled designs is needed. *E*‐values can also be calculated for both CCO and SCCS [62], although it is important to note that they would quantify the strength of time‐varying unmeasured confounding. Negative controls [63] are typically used to detect unmeasured confounding—which in the self‐controlled setting is time‐varying. It is therefore important to choose a negative control for which all assumptions of the self‐controlled design in question hold, and that is hypothesised to be subject to the same underlying—time‐varying—confounding that is causing concern for the primary analyses. These criteria are more stringent than for between‐person designs and might make it challenging to identify suitable negative outcome controls for self‐controlled analyses. Negative exposure controls, specifically, active comparators—are more straightforward to identify and apply in these designs, and detailed guidance on how to use these to control for time‐varying confounding has been published [10, 51].

## 7 Conclusions and Future Directions

Self‐controlled designs can be a useful complement to traditional between‐person designs when strong unmeasured, time‐stable confounding is a concern. They remain an area of active research, with several extensions and new tools for implementation recently developed. Further work on the use of sensitivity analysis such as QBA in the self‐controlled setting would be of value, and the increasing use of the target trial [64] and estimand frameworks [65] highlights the importance of theoretical work formalising the assumptions underlying self‐controlled designs to facilitate their interpretation. Despite the strong assumptions they rely on, self‐controlled designs are a useful complement to more traditional designs which can support triangulation of different lines of evidence, ultimately allowing for more robust inferences around drug safety.

## Conflicts of Interest

The authors declare no conflicts of interest.

## Supporting information


Data S1.

Data S2.

Data S3.
