# Supplementary material for: Core Concepts: Self‐Controlled Designs in Pharmacoepidemiology
Source: Pharmacoepidemiol Drug Saf. 2025 Jan 13;34(1):e70071. doi: 10.1002/pds.70071 (PMC11729261; doi:10.1002/pds.70071)
Supplement: Supplementary file 2 — Data S2. [file PDS-34-e70071-s001.docx]

## Annotated reference list

This annotated reference list serves to highlight and recommend resources which we have found particularly helpful ourselves when implementing self-controlled designs for the first time. It includes previously published reviews, papers and books on self-controlled methods, but also tutorials and tools to implement the designs.

Background reading

We refer readers who are relatively new to unmeasured confounding (or wish to refresh their knowledge) to the review of Uddin and colleagues (65) that covers a wide range of pharmacoepidemiological methods to address unmeasured confounding, including self-controlled designs. For a general introduction to self-controlled designs in pharmacoepidemiology, we recommend Hallas and Pottegård (4), which provides a general overview, and Cadarette and colleagues which introduces ISPE-endorsed terminology for self-controlled designs (6). Additionally, this overview paper by Mostofsky and colleagues may be of interest (66). To get an idea of how self-controlled designs have been used in pharmacoepidemiology, we recommend the reviews by Gault and colleagues (67) and Nordmann and colleagues (68).

Outcome-anchored designs

The Open Access Methods primer by Lewer and colleagues (24) provides a concise introduction to case-crossover designs. A more comprehensive background is provided by Maclure and Mittleman (2) in a paper we found very useful while writing this Core Concepts paper. Mittleman and colleagues’ (11) eminently readable paper puts case-crossover and case-time-control studies into a causal inference framework, later formalised by Shahn and colleagues (51).

Sample R code for the data management analysis of a case crossover study has been published by Zhang (69), although there are many possible ways of approaching this task. R Code from the OHDSI collaboration is also available showing the implementation of both a case-crossover and case-time control study (https://github.com/OHDSI/CaseCrossover).

Exposure-anchored designs

The Open Access Research Methods & Reporting paper by Petersen and colleagues (34) provides a good overview of the self-controlled case series design and how to deal with violations of its assumptions. We also recommend the excellent tutorial by Whitaker and colleagues for those considering setting up their first self-controlled case series study or those wishing to learn about the method in detail (3). Readers interested in the proofs behind many of the self-controlled case series’ concepts and analytical extensions may be interested in the work by Whitaker and colleagues, including (36, 37). Readers wanting to apply the self-controlled cases series to study vaccine safety can use Weldeselassie and colleagues’ (70) review for best practice recommendations.

The SCCS R package conducts both data management and analysis and is available on CRAN (https://cran.r-project.org/package=SCCS) with an accompanying, detailed, book (71). Alternative code from the OHDSI collaboration is also available (https://github.com/OHDSI/SelfControlledCaseSeries). Sample code in Stata and SAS is provided on the self-controlled case series webpage (https://sccs-studies.info/index.html). It is worth noting that there is no existing PROC for fitting a conditional Poisson regression available in SAS; users of this software may instead rely on user-written macros (https://sccs-studies.info/index.html), or, they could include the person ID as a fixed effect in a standard Poisson regression, although this will be very inefficient if the case series is large.

References

66. Uddin MJ, Groenwold RH, Ali MS, de Boer A, Roes KC, Chowdhury MA, Klungel OH. Methods to control for unmeasured confounding in pharmacoepidemiology: an overview. Int J Clin Pharm. 2016;38(3):714-23.

67. Gault N, Castañeda-Sanabria J, De Rycke Y, Guillo S, Foulon S, Tubach F. Self-controlled designs in pharmacoepidemiology involving electronic healthcare databases: a systematic review. BMC Med Res Methodol. 2017;17(1):25.

68. Nordmann S, Biard L, Ravaud P, Esposito-Farèse M, Tubach F. Case-Only Designs in Pharmacoepidemiology: A Systematic Review. PLOS ONE. 2012;7(11):e49444.

69. Zhang Z. Case-crossover design and its implementation in R. Ann Transl Med. 2016;4(18):341.

70. Weldeselassie YG, Whitaker HJ, Farrington CP. Use of the self-controlled case-series method in vaccine safety studies: review and recommendations for best practice. Epidemiol Infect. 2011;139(12):1805-17.
